# Cancer-Associated Fibroblasts Suppress Cancer Development: The Other Side of the Coin

**DOI:** 10.3389/fcell.2021.613534

**Published:** 2021-02-04

**Authors:** Zhanhuai Wang, Qi Yang, Yinuo Tan, Yang Tang, Jun Ye, Bin Yuan, Wei Yu

**Affiliations:** ^1^Department of Colorectal Surgery and Oncology, Key Laboratory of Cancer Prevention and Intervention, Ministry of Education, The Second Affiliated Hospital, Zhejiang University School of Medicine, Hangzhou, China; ^2^Department of Pathology, The Second Affiliated Hospital, Zhejiang University School of Medicine, Hangzhou, China; ^3^Department of Medical Oncology, Key Laboratory of Cancer Prevention and Intervention, Ministry of Education, The Second Affiliated Hospital, Zhejiang University School of Medicine, Hangzhou, China; ^4^Department of Gastroenterology, The Second Affiliated Hospital, Zhejiang University School of Medicine, Hangzhou, China; ^5^Department of Biochemistry and Molecular Medicine, School of Medicine and Health Sciences, The George Washington University, Washington, DC, United States; ^6^Department of Radiation Oncology, Key Laboratory of Cancer Prevention and Intervention, Ministry of Education, The Second Affiliated Hospital, Zhejiang University School of Medicine, Hangzhou, China

**Keywords:** cancer-associated fibroblasts, neoplasms, tumor microenvironment, biomarker, Humans

## Abstract

Cancer-associated fibroblasts (CAFs) are the main stromal components of cancer, representing a group of heterogeneous cells. Many studies indicate that CAFs promote tumor development. Besides, evidence of the tumor suppression effects of CAFs keeps on merging. In the tumor microenvironment, multiple stimuli can activate fibroblasts. Notably, this does not necessarily mean the activated CAFs become strong tumor promoters immediately. The varying degree of CAFs activation makes quiescent CAFs, tumor-restraining CAFs, and tumor-promoting CAFs. Quiescent CAFs and tumor-restraining CAFs are more present in early-stage cancer, while comparatively, more tumor-promoting CAFs present in advanced-stage cancer. The underlying mechanism that balances tumor promotion or tumor inhibition effects of CAFs is mostly unknown. This review focus on the inhibitory effects of CAFs on cancer development. We describe the heterogeneous origin, markers, and metabolism in the CAFs population. Transgenetic mouse models that deplete CAFs or deplete CAFs activation signaling in the tumor stroma present direct evidence of CAFs protective effects against cancer. Moreover, we outline CAFs subpopulation and CAFs derived soluble factors that act as a tumor suppressor. Single-cell RNA-sequencing on CAFs population provides us new insight to classify CAFs subsets. Understanding the full picture of CAFs will help translate CAFs biology from bench to bedside and develop new strategies to improve precision cancer therapy.

## Introduction

Fibroblasts are one of the significant stromal components in many organs, e.g., the gastrointestinal tract. They participate in adjacent tissue components’ function through paracrine signaling and juxtacrine signaling (Biswas et al., 2015; Kalluri, 2016). One of their most crucial tasks is regulating ECM synthesis. When tissue injury happens, fibroblasts are getting ready to respond. They actively act to restrain the injury place and reconstruct the wound tissue framework. The classic description of cancer is “wounds that never heal.” Could fibroblasts act as potential defenders against cancer progression? Numerous studies are being carried out to answer this question, but the results are unclear or sometimes contradicting. Generally, fibroblasts observed within and adjacent to the cancer are termed as cancer-associated fibroblasts (CAFs). In practice, the criteria to define CAFs are (1) mesenchymal cell isolated from a tumor; (2) negative markers for epithelial cells, endothelial cells and leukocyte markers; (3) elongated morphology; and (4) exclude the mutations of cancer cells (Sahai et al., 2020). CAFs are a group of heterogeneous cells and are the most prevalent stromal components of the tumor microenvironment. Previous studies have focused on tumor cells, yet now, it is increasingly recognized that the stromal components of the tumor microenvironment play critical roles in cancer progression (Hanahan and Weinberg, 2011). Importantly, CAFs play diverse parts in the interaction between different components in the tumor microenvironment. Thus, in cancer biology, understanding the functional evolution of CAFs during cancer development becomes a fundamental question to answer.

**FIGURE 1:**
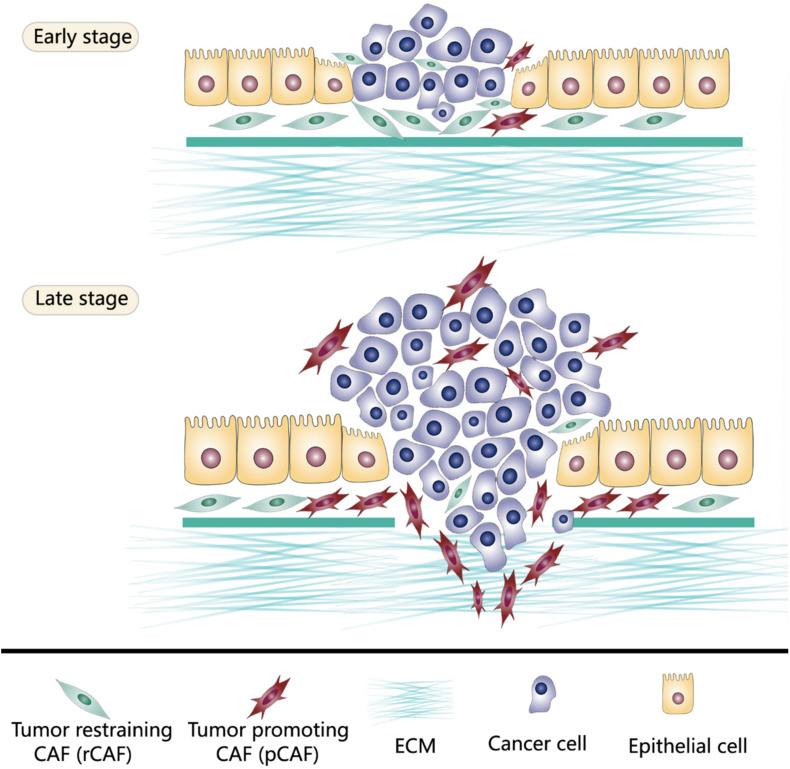
Tumor-restraining CAFs (rCAFs) and tumor-promoting CAFs (pCAFs) both exist in the tumor microenvironment. rCAFs are frequently present in early-stage cancer which protect normal tissue against cancer invasiveness. However, in advanced-stage disease, tumor cells reprogram CAFs through continuous messages exchange to build cancer supporting stromal niche. Thus, pCAFs are predominantly present in late-stage cancer.

**FIGURE 2:**
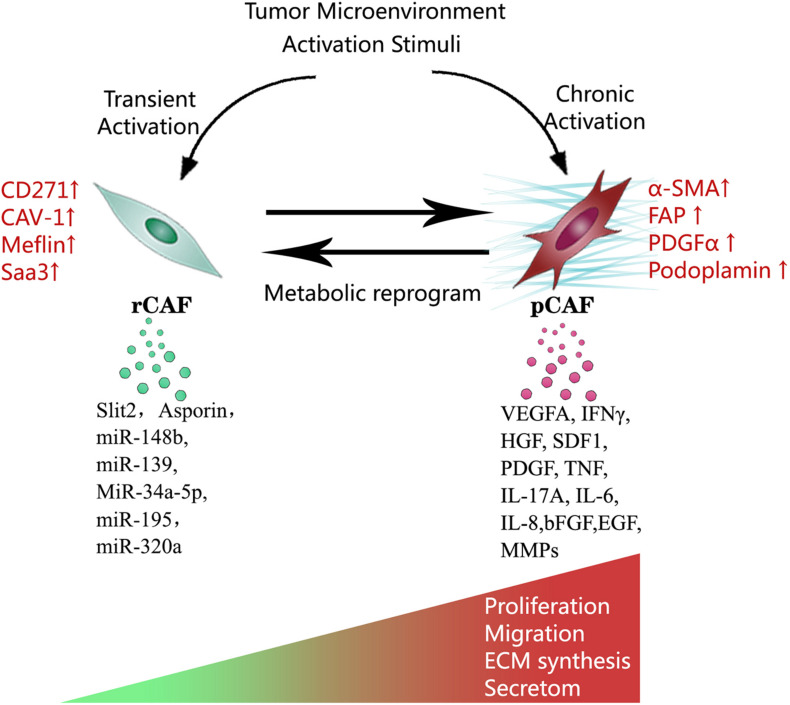
Multiple stimuli in the tumor microenvironment activate CAFs. Notably, this activation process does not necessarily mean the activated CAFs become strong cancer promoters immediately. Depending on the degree of activation, CAFs can be functionally divided into tumor-restraining CAFs, and tumor-promoting CAFs. Transient activation makes tumor-restraining CAFs and chronic activation makes tumor-promoting CAFs. Tumor-restraining CAFs are morphologically similar to quiescent fibroblasts. Tumor-promoting CAFs undergo morphological changes from spindle-shaped into cruciform or stellate shaped. Two types of CAFs show the main difference in cellular markers, metabolic status, proliferation, migration, ECM synthesis, and secretory phenotype.

In the tumor microenvironment, potential stimuli that activate fibroblasts include bone morphogenetic protein (BMP), interleukin-1, interleukin-6, platelet-derived growth factor (PDGF), sonic hedgehog (SHH), reactive oxygen species (ROS), transforming growth factor-β (TGF-β), as well as TNF. These stimuli are released by tumor cells and multiple stromal components, including CAFs themselves (Theiss et al., 2005; Erez et al., 2010; Kalluri, 2016). The activated fibroblasts specifically express α smooth muscle actin (α-SMA), fibroblast activation protein (FAP), fibroblast-specific protein 1 (FSP1), PDGF receptor-β (PDGFRβ), and some other myofibroblasts markers. α-SMA, a cytoskeleton protein, functions as a cell contraction regulator, is most commonly used as an activated fibroblast marker (Ohlund et al., 2014; Kalluri, 2016; Sahai et al., 2020). Cell morphology is a reliable way to distinguish activated CAFs within the quiescent fibroblasts. Activated CAFs undergo morphological changes from spindle-shaped into cruciform or stellate shaped. They become more proliferative, migrative, and metabolically active, with the enhanced acquisition of ECM production and synthetic phenotype. They have different epigenetic expression pattern compared to quiescent fibroblasts (Albrengues et al., 2015; Zhang et al., 2015; Kalluri, 2016). Notably, the activation of fibroblasts by stimuli in the tumor microenvironment does not necessarily mean these activated CAFs become strong cancer promoters immediately. Indeed, the varying degree of CAFs activation patterns produce quiescent CAFs, tumor-restraining CAFs, and tumor-promoting CAFs (Sugimoto et al., 2006; Kobayashi et al., 2019). Quiescent CAFs and tumor-restraining CAFs are frequently present in early-stage cancer, while comparatively, more tumor-promoting CAFs are predominantly present in advanced-stage disease. The chronic activation of CAFs requires continuous cross-talk between CAFs and cancer cells. This permanent cross-talk educates CAFs to accquire the pro-tumorigenic ability. Meanwhile, the stimuli that promote CAFs activation are essential factors that enhance tumor niche formation (Roberts et al., 2017). The co-evolution of CAFs and cancer cells continues throughout all the cancer progression stages (Figures 1, 2).

The tumor microenvironment plays a key part in fostering tumor progression through the collaboration of multiple components. Besides, evidence of the tumor inhibition effects of individual cancer stromal component keeps on merging. The underlying mechanism that balances tumor promotion or tumor inhibition effects of the tumor microenvironment is mostly unknown. Most studies that explore CAFs functions show that CAFs support tumor growth. Naturally, the reverse side evidence of this is much less. If the tumor stroma inhibiting effect continuously exists, the tumor can not necessarily develop. Failure to educate quiescent fibroblasts to acquire pro-tumorigenic ability means tumor cells might not survive (Mueller and Fusenig, 2004; Proia and Kuperwasser, 2005). In the very beginning of tumorigenesis, tumor cells conduct a dedicated education system to reprogram CAFs and other tumor stroma cells. In the later stage, tumor cells deliver messages to distant healthy organs stroma to build a pre-metastatic stromal niche (Hanahan and Weinberg, 2011). Recently, tumor associated-extracellular vesicles (EVs) have been identified as an important cellular interchange mechanism between tumor cells and CAFs (Becker et al., 2016; Maacha et al., 2019). EVs isolated from tumor cells and CAFs are implicated in multi-steps of CAFs evolution, such as normal fibroblasts (NFs) differentiation into CAFs, CAF-like state maintenance, and CAFs’ function support (Luga et al., 2012; Webber et al., 2015; Richards et al., 2017; Giusti et al., 2018).

Understanding the time point when CAFs shift from tumor defender into tumor supporter is critically essential. Thus, preventing CAFs functional change from a tumor-supportive phenotype will be potentially promising in anti-tumor therapy. This review focus on the inhibitory effects of CAFs on cancer development. We describe the heterogeneous origin, markers, and metabolism in the CAFs population. Transgenetic mouse models that deplete CAFs or deplete CAFs activation signaling in the tumor stroma present direct evidence of CAFs’ protective effects against cancer. Moreover, we outline CAFs subpopulation and CAFs derived soluble factors that act as a tumor suppressor. Single-cell RNA-sequencing on CAFs population provides us new insight to classify CAFs subsets. The main findings concerning the tumor inhibitory effects of CAFs are list in Table 1.

**TABLE 1:** Studies focus on the tumor inhibitory effects of CAFs.

Author	Cancer type	Main findings
Dai et al., 2019	Mice pancreatic cancer	1 In RAMP3-/- mice, spleen injection of PAN02 murine pancreatic cancer cells showed reduced liver metastasis. 2 RAMP3-/- mice metastatic tumor showed decreased podoplanin positive or α-SMA positive CAFs. 3 Primary RAMP3-/- CAFs inhibited proliferation, migration, and metastasis in co-cultures with PAN02 murine pancreatic cancer cells.
Mizutani et al., 2019	Mice Pancreatic ductal adenocarcinoma (PDAC)	1 Meflin is expressed in CAFs originate from pancreatic stellate cells 2 Meflin positive CAFs showed decreased a-SMA expression and stromal collagen regulating 3 Meflin negative CAFs showed more aggressive pro-tumorigenic functions 4 Meflin knockout mice showed a poorly differentiated tumor with more a-SMA + CAFs compared to Meflin wild type mice
Djurec et al., 2018	Mice PDAC	1 PDGFRα + Saa3 + CAFs stimulated mice PDAC growth, but PDGFRα + Saa3- CAFs inhibited tumor growth. 2 The PDGFRα + Saa3- CAFs inhibited tumor growth by overexpression Mpp6
Gerling et al., 2016	Mice colitis associated-colon cancer (CAC)	1 Downstream Hh signaling is restricted to the tumor stroma particularly in CAFs 2 Hh signaling deletion in CAFs promoted tumorigenesis, whereas Hh activation inhibited tumor progression 3 Hh signaling in CAFs suppressed tumor by regulating BMP activity and inhibting colonic stem cell signature
Maris et al., 2015	Breast cancer	1 Asporin suppressed TGF-β1-mediated SMAD2 phosphorylation, EMT, and cancer stem cell signature. 2 Asporin overexpression CAFs reduce tumor growth in the mice xenograft TNBC model
Koliaraki et al., 2015	Mice CAC	Genetic deletion of IKKβ in COLVI + CAFs caused decreased tumor growth and inflammation in the CAC mice model. The effect is mainly due to the down-regulated IL-6 release in IKKβ-deficient CAFs compared to control
Pallangyo et al., 2015	Mice CAC	1 Genetic deletion of IKKβ in COL1A2 + CAFs in mice colon cancer accelerated tumor growth 2 IKKβ-deficient COL1A2 + CAFs showed enhanced secretion of HGF, which promoted tumor growth through HGF–Met signaling
Ozdemir et al., 2014	Mice PDAC	1 Effects of genetic a-SMA + myofibroblast depletion were test in both early and late stage PDAC 2 a-SMA + myofibroblast depletion tumor showed more invasive, undifferentiated, and necrotic characteristics with a poor survival compared to control.
Rhim et al., 2014	Mice PDAC	1 SHH-deficient PDAC showed decreased a-SMA-positive myofibroblasts compared to the control tumor. 2 SHH-deficient PDAC exhibited undifferentiated histology, increased proliferation, vascularity, and reduced survival time.
Zhang et al., 2013	Mice skin fibrosarcoma	1 FSP1 was predominantly expressed in procollagen I + fibroblasts in mice skin 2 Genetic deletion of FSP1 + CAFs enhanced mice skin fibrosarcoma formation
Chang et al., 2012	Breast cancer	1 CAFs derived Slit2 conducted its tumor inhibition effects by bind to Robo1 receptor expressed in cancer cells.

***Abbreviations:** CAC, colitis associated-colon cancer; Evs, extracellular vesicles; HGF, hepatocyte growth factor; HR, hormone receptor; OSCC, Oral squamous cell carcinoma; PDAC, Pancreatic ductal adenocarcinoma; and TNBC, triple-negative breast cancer.*

## The Heterogeneity of CAFs Population

### Heterogenous CAFs Origin

Despite the highly organized NFs, the CAFs population is highly heterogeneous. The origin of CAFs is still contested. The direct correlation between CAFs subtype function and CAFs subtype cellular origin is not clearly illustrated. However, it is convincing that CAFs originated from different cell lineages might have distinct functional phenotypes (Ishii et al., 2015; Sahai et al., 2020). In general, CAFs have four main sources of cellular origin. The primary source is normal local fibroblasts, which are activated by stimuli from the tumor microenvironment. Mesenchymal stem cells (MSCs) and other mesenchymal precursor cells are other sources. They are recruited to the TME to become CAFs-state cells by cytokines and chemokines, including TGF-β and CXC-chemokine ligand 12 (CXCL12; Koliaraki et al., 2017). Endothelial cells and epithelial cells do not belong to the fibroblast lineage, but they could transdifferentiate into CAFs-state cells. Finally, a self-renewable CAFs-stem cell population might exist in the hierarchical organization, and these cells share similar characteristics as MSCs. They differentiate into progeny CAFs including cancer-promoting CAFs and cancer-restraining CAFs (Worthley et al., 2015).

### Heterogenous CAFs Marker

Studies to identify CAF subsets are still in their infancy. In the experiment, CAFs are isolated through flow cytometry-based cell sorting by using a series of combined markers. The markers that CAFs lack expression of are CD31 (an endothelial marker), CD45 (a hematopoietic cell marker), desmin (a smooth muscle cell marker), and EPCAM (epithelial cell adhesion molecule, an epithelial cell marker). These markers are combined with a representative CAFs marker (e.g., FAP) for CAFs sorting (Feig et al., 2013; Calon et al., 2015; Costa et al., 2018). The most commonly used CAFs markers include but are not limited to α-SMA, Collegen1A1, FAP, FSP1, PDGFRα and PDGFRβ, Podoplanin, and vimentin. However, all of these markers are not CAFs specific and can be expressed in other cell types in cancer or normal tissues (Kalluri, 2016). α-SMA is unable to identify all CAFs in the TME, and it is expressed in smooth muscle in normal gastrointestinal and vascular (Koliaraki et al., 2017; Ohlund et al., 2017). FAP combined with CD45 is used to label a subgroup of cancer-associated macrophages (Arnold et al., 2014). FSP1 also corresponds to epithelial cells experiencing epithelial-to-mesenchymal transition (EMT; Rhim et al., 2012; Fischer et al., 2015). In a liver fibrosis model, FSP1 distinguishes inflammatory macrophage subpopulation (Osterreicher et al., 2011). The different origins of CAFs add to the complexity of CAFs definitions. A sole CAFs marker is impossible to find theoretically. Depending on different markers used, different results regarding CAFs pro-tumorigenic function (pCAFs) or CAFs tumor-restain (rCAFs) function can be obtained (Kobayashi et al., 2019). One of the most urgent works required in CAFs biology is to explore the different markers of subtype CAFs based on biology and function.

### Heterogenous CAFs Metabolism

Traditionally, the glycolytic pathway has been considered the main metabolic pathway in cancer. Recent studies revealed that cancers are highly organized tissues and are categorized with heterogeneous metabolism in different components (Wallace, 2012). Even in the CAFs population, the heterogeneous metabolic status also exists. Compared to quiescent fibroblasts, parts of activated CAFs undergo metabolic changes and become catabolic CAFs. Biomarkers of this catabolic CAFs phenotype including down-regulated caveolin-1 (Cav-1; Martinez-Outschoorn et al., 2011) and up-regulated monocarboxylate transporter 4 (MCT4; Whitaker-Menezes et al., 2011). Cav-1 is abundantly expressed in NFs. Loss of Cav-1 expression is an autophagy marker, indicating decreased mitochondrial metabolisms, such as oxidative phosphorylation (OXPHOS), increasing glycolysis and oxidative stress (Sotgia et al., 2011). MCT4, with its expression controlled by HIF-1, is the main cellular lactate exporter (Ullah et al., 2006). Recent studies demonstrate the collaboration of catabolic fibroblasts and anabolic cancer cells, which is the so-called metabolic coupling. This metabolic coupling exists in many different human malignancies, such as breast cancer, head and neck cancer, and prostate cancer (Giatromanolaki et al., 2012; Witkiewicz et al., 2012; Curry et al., 2013). The driving pathway of catabolic CAFs are HIF1-α, NFκB signaling, and TGF-β signaling, promoting autophagy, glycolysis, oxidative stress, and senescence (Martinez-Outschoorn et al., 2014). These catabolic CAFs generate local mitochondrial fuels, such as fatty acids, glutamine, ketone bodies, lactate, to support the cancer microenvironment (Martinez-Outschoorn et al., 2014). Noticed that not all the CAFs become catabolic CAFs, the catabolic CAFs and the anabolic CAFs both exist. The catabolic changes of CAFs could be reversed upon anti-oxidants treatments (Yang et al., 2016; Monti et al., 2017; Zhang et al., 2018). Does the transformation from tumor-restraining CAFs into tumor-promoting CAFs accompany with catabolic phenotype changes? The answer is still not clear. Explore the heterogeneity in CAFs metabolism will help us to classify CAFs in a critical functional aspect.

## The Protective Action of CAFs Against Cancer: Evidence From Transgenic Mice

### Depletion of CAFs Enhances Tumor Development

The transgenic mouse model allows us to deplete CAFs through germline mutation or organ-specifically in mature tissue. Such a model helps to investigate roles that CAFs play in different stages of carcinogenesis. What happens if CAFs are diminished in certain types of tumors? Results of recent studies strikingly indicated that CAFs depletion causes rapid tumor progression rather than tumor suppression. a-SMA is one of the most commonly used CAFs markers, distinguishing activated fibroblasts from the quiescent fibroblasts in tumor microenvironments (Kalluri, 2016). Ozdemir et al., applied a genetic approach to deplete a-SMA + myofibroblasts in mice selectively. They aimed to examine the function of a-SMA + myofibroblasts in early-stage, as well as late-stage pancreatic ductal adenocarcinoma (PDAC). They crossed a-SMA- thymidine kinase (TK) transgenic mice that allowed target depletion of a-SMA + myofibroblasts and the mice that developed spontaneous PDAC. By crossed two type of mice, a-SMA + myofibroblasts could be selectively depleted in PDAC tissue. The effects of a-SMA + myofibroblasts depletion were analyzed in both early-stage and late-stage PDAC. Compared to control, a-SMA + myofibroblast depletion tumor was significantly more invasive, undifferentiated, and necrotic. Mice bearing a-SMA + myofibroblast depletion tumor ended up with multiple adverse outcomes. The author also suggested that a-SMA + myofibroblast produced collagen I and the associated fibrosis protected the host against tumorigenesis at both early and late stages of PDAC (Ozdemir et al., 2014). Zhang et al. tested the hypothesis of whether depletion FSP1 fibroblasts impair skin fibrosarcoma formation. The author applied FSP-TK transgenic mice, which allowed FSP1 + cells to be selectively depleted upon administration of ganciclovir (GCV; Salomon et al., 1995; Iwano et al., 2001). Zhang et al. found that FSP1 was predominantly expressed in procollagen I + fibroblasts in mice skin. Thus, FSP1 + depletion in stromal cells mainly caused CAFs depletion. Mice model of skin fibrosarcoma was induced via carcinogen methylcholanthrene (MCA) subcutaneous injection. A Ablation of FSP1 + cells enhanced tumor formation and altered skin fibrosarcoma morphology. Interestingly, these skin tumors showed epithelial phenotype instead of fibroblastoid. The author also showed that degrading collagen produced by fibroblasts promoted tumor formation in the long-term “tumor-free” mice. These findings indicated that FSP1 + CAFs and collagens exert a critical protective role against chemical induced fibrosarcoma in the skin (Zhang et al., 2013).

### Depletion of Activation Signaling in CAFs Enhances Tumor Progression

Cancer-associated fibroblasts activation signaling relies on various stimuli from the TME, and SHH is one of these. SHH, a soluble ligand of hedgehog signaling, is frequently overexpressed by neoplastic cells, which stimulates CAFs to form a fibroblast-rich desmoplastic stroma (Valenti et al., 2017). Earlier studies indicated that over activation of hedgehog-signaling accelerated tumorigenesis in the pancreatic epithelium (Mao et al., 2006; Pasca Di Magliano et al., 2006; Morton et al., 2007). Since the deletion of SHH expression might impair CAFs activation signaling, what is the impact on malignant progression if SHH expression is removed from cancer cells? Rhim et al used the transgenic mice to delete SHH in the PDAC model. The SHH-deficient tumor showed decreased stromal content, especially a-SMA-positive myofibroblasts, compared to the control tumor. These tumors exhibited more aggressive features with undifferentiated histology, increased proliferation, and vascularity. SHH deletion also caused more frequent acinar-to-ductal metaplasia (ADM) and pancreatic intraepithelial neoplasia (PanIN) at a younger age. Thus, SHH deletion lead to rapid death in mice. SHH deletion in pancreatic cancer showed increased Zeb1 and Slug expression consistent with EMTs and increased metastasis (Rhim et al., 2014). Similar results were reported in a mouse model of colitis-associated colonic tumorigenesis. In that study, Gerling et al applied hedgehog (Hh) signaling reporter mice. They demonstrated that Hh signaling deletion promoted tumorigenesis, whereas stroma-specific Hh activation significantly attenuated tumor progression. Activated Hh signaling in CAFs suppressed tumor growth through regulating BMP activity and inhibiting colonic stem cell gene expression (Gerling et al., 2016). The IKKβ-dependent NF-κB signaling activation is considered as a key to connect inflammation and carcinogenesis (Greten et al., 2004). CAFs from the cervical, mammary, pancreatic, and skin tumors exhibit a proinflammatory signature regulated by NF-κB signaling (Erez et al., 2010). Two separate studies probed the effects of IKKβ on CAFs during tumor development. The first study used Tg (CollagenVI-Cre) mice, which allowed tracing of the distribution of collagen type VI positive (COLVI +) fibroblasts in the mice intestine. Tg (CollagenVI-Cre) mice were crossed with Ikkβ^F/F^ mice to generate IKKβ deficient COLVI + fibroblasts. These transgenic mice were further applied with AOM/DSS to form colitis-associated cancer (CAC). IKKβ deficiency in COLVI + CAFs caused decreased tumor growth and inflammation in the CAC mice model. The effect is mainly due to the down-regulated IL-6 release in IKKβ-deficient CAFs compared to control (Koliaraki et al., 2015). The second study, however, showed the tumor-restraining function of IKKβ/NF-κB in CAFs. Koliaraki et al used a different Cre, collagen type a2 Cre (Col1a2-creER), to trace fibroblasts. And IkkβF/F mice were crossed with Col1a2-creER mice. COL1A2 + fibroblasts represent a larger population than COLVI + fibroblasts. The genetic deletion of IKKβ in COL1A2 + CAFs in the similar CAC model unexpectedly accelerated tumor growth. The Ikkβ deficient CAFs showed impaired Smad7 and Smurf1, which were both TGF-β pathway negative regulators. Thus, the upregulation of TGF-β signaling was observed in Ikkβ deficient CAFs. Moreover, these CAFs showed enhanced secretion of hepatocyte growth factor (HGF), which promoted tumor growth through HGF–Met signaling (Pallangyo et al., 2015). Since COL1A2 targets nearly 80% of PDGFRα + fibroblasts, COL1A2 + fibroblasts comprised a much larger fibroblasts population than COLVI + fibroblasts. The possible explanation of conflict results is that different fibroblast subpopulations may exhibit distinct IKKβ/NF-κB signaling functions. Meanwhile, CAFs display different activation status could display different NF-κB signaling status.

## CAFs Subpopulation Inhibit Tumor Progression

Despite numerous results indicating CAFs’ pro-tumorigenic effects, here, we provide shreds of evidence that CAFs subsets restrain tumor progression. Djurec et al., showed that PDGFRα + CAFs derived from a PDAC mouse model promoted tumor cell growth, and that normal pancreatic fibroblasts inhibited tumor growth. When PDGFRα + CAFs were subdivided by the expression of Saa3, a protein belonging to the acute-phase serum amyloid A (SAA) apolipoprotein family, PDGFRα + Saa3- CAFs and PDGFRα + Saa3 + CAF showed different properties. PDGFRα + Saa3 + CAFs stimulated mice PDAC growth in both the orthotopic model and organoid cultures; however, PDGFRα + Saa3- CAFs inhibited tumor growth. The PDGFRα + Saa3- CAFs exerted their tumor inhibition effects by overexpressing membrane palmitoylated protein 6 (Mpp6). This study sheds light on the future direction of target therapy through the management of Mpp6 expression (Djurec et al., 2018). Dai et al found that RAMP3 deficient CAFs inhibited tumor growth and metastasis. RAMPs, receptor-activity-modifying proteins, are modulators of G-protein-coupled receptors, which function as tumor angiogenesis regulator and prognostic marker (Mclatchie et al., 1998; Fang et al., 2018; Mackie et al., 2019). In RAMP3-/- mice, spleen injection of PAN02 murine pancreatic cancer cells showed significantly reduced liver metastasis. Compared to wild-type mice, liver metastatic lesion in RAMP3-/- mice showed decreased podoplanin positive or α-SMA positive CAFs. Moreover, primary cultured RAMP3-/- CAFs inhibited proliferation, migration, and metastasis in co-cultures with PAN02 murine pancreatic cancer cells *in vitro* and *in vivo* (Dai et al., 2019). Meflin is a mesenchymal stromal/stem cell marker and indicates their undifferentiated state. In pancreatic cancer, Meflin is expressed in CAFs originate from pancreatic stellate cells. Meflin positive CAFs showed decreased a-SMA expression and stromal collagen regulation, while Meflin negative CAFs showed more aggressive pro-tumorigenic functions. In the mouse PDAC model, Meflin knockout mice showed a poorly differentiated tumor with more a-SMA + CAFs compared to Meflin wild type mice. Results suggested Meflin served as a tumor-restraining CAFs marker in PDAC (Mizutani et al., 2019).

## CAFs Derived Soluble Factors Suppress Tumor Progression

Cancer-associated fibroblasts support cancer progression by secreting various soluble factors, whereas CAFs can also derive tumor restraining factors. In breast cancer, CAFs derived Slit2 acted as a tumor inhibitor. Slit2 exerted tumor suppression effects by binding to Robo1 receptor expressed in cancer cells. Stable Robo1-depletion in breast cancer cells abolished the CAFs tumor suppression effect in the orthotopic mouse model. Meanwhile, ectopic Robo1 overexpression in breast cancer cells enhanced CAFs related tumor suppression effect. The active Slit2/Robo1 signaling prevented β-catenin translocation into nuclei, which resulted in c-myc and cyclin D1 downregulation through the PI3K/Akt pathway (Chang et al., 2012). Asporin, a stromal secreted extracellular matrix protein, inhibited canonical TGF-β/Smad signaling by binding to TGF-β1 (Kizawa et al., 2005). The expression of asporin in CAFs increased when exposed to gastric cancer cells. CAFs-derived asporin activated CD44-Rac1 pathway in cancer cells and coordinated the co-invasion of these two types of cells (Satoyoshi et al., 2015). Interestingly, asporin functional studies in breast cancer is another story. Triple-negative breast cancer (TNBC) cells suppressed CAFs’ asporin expression by secreting IL-1β, while hormone receptor (HR) positive breast cancer induced CAFs asporin expression. *In vitro* studies on breast cancer cells indicated asporin suppressed TGF-β1-mediated SMAD2 phosphorylation, EMT, and cancer stem cell signature. In the murine model of TNBC, asporin overexpression CAFs could significantly reduce tumor growth. The results of the two studies seem to conflict with each other. TGF-β signaling has both tumor suppression and tumor promotion effects depending on the cancer type and cancer stage (Seoane and Gomis, 2017). Asporin is a TGF-β1 natural inhibitor, which might also have various services in different cancer types.

MicroRNAs are known as small molecular RNA, which bind to their target mRNAs and negatively modulate gene expression at the post-transcriptional level. MicroRNAs transferred by exosomes are a common way of communication connecting CAFs and tumor cells (Li et al., 2018). Recently studies investigated the microRNA changes and their function in CAFs derived exosomes. Our previous review illustrates CAFs-derived microRNAs promote cancer development through a variety of approaches (Wang et al., 2017). During tumor progression, CAFs secreted tumor-promoting microRNAs show increased expression and CAFs secreted tumor-suppressing microRNAs are inhibited (Wang et al., 2017). Several studies indicated that tumor-suppressing microRNAs could strongly inhibit tumor development when they were re-expressed in CAFs. These CAFs derived tumor suppressor microRNAs include miR-195 in cholangiocarcinoma (Li L. et al., 2017), MiR-34a-5p in oral squamous cell carcinoma (OSCC; Li et al., 2018), miR-148b in endometrial cancer (Li et al., 2019), miR-139 in gastric cancer (Xu et al., 2019), and miR-320 in hepatocellular carcinoma (HCC; Zhang et al., 2017; Table 2).

**TABLE 2:** CAFs derived tumor suppression microRNAs.

Author	Cancer type	Main findings
Li et al., 2019	Endometrial cancer	1 MiR-148b decreased in CAFs and CAFs-derived exosomes compared to NFs 2 CAFs derived miR-148b transferred to endometrial cancer cells by exosomes 3 MiR-148b overexpression in CAFs suppressed endometrial cancer metastasis *in vitro* and *in vivo* by directly binding to DNMT1
Xu et al., 2019	Gastric cancer	1 MiR-139 level was down-regulated in tumors compared with adjcent normal tissues 2 Exosomal miR-139 in CAFs was reduced compared to NFs 3 Exosomes shuttled miR-139 from fibroblasts to cancer cells 4 MiR-139 overexpression in CAFs suppressed cancer cells growth and metastasis by inhibiting the expression of MMP11
Li et al., 2018	OSCC	1 MiR-34a-5p in CAF-derived exosomes was reduced compared to NFs 2 Fibroblasts derived exosomal miR-34a-5p could transfer to OSCC cells 3 miR-34a-5p overexpression in CAFs inhibited OSCC cells proliferation and metastasis by binding to AXL in cancer cells
Li L. et al., 2017	Cholangiocarcinoma	1 Cholangiocarcinoma cells and the adjoining CAFs showed down-regulated miR-195 2 EVs shuttled miR-195 from fibroblasts to cancer cells 3 miR-195 overexpression in fibroblasts suppressed growth and invasion of cholangiocarcinoma cells 4 miR-195 loaded EVs inhibit tumor and improve survival of in a rat model of cholangiocarcinoma.
Zhang et al., 2017	HCC	1 miR-320a level was reduced in CAFs-derived exosomes compared to NFs 2 Fibroblasts derived exosomal miR-320a could transfer to HCC cells 3 Exosomal miR-320a binded to PBX3 in HCC cells and inhibited their proliferation and metastasis. 4 MiR-320a-PBX3 axis suppressed tumor progression by inhibiting MAPK pathway acitivation

***Abbreviations:** DNMT1, DNA (cytosine-5)-methyltransferase 1; Evs, extracellular vesicles; HCC, hepatocellular carcinoma; HGF, hepatocyte growth factor; HR, hormone receptor; OSCC, Oral squamous cell carcinoma; and TNBC, triple-negative breast cancer.*

## CAFs Related Protein Provide Favorable Clinical Outcomes

Many studies have addressed the clinical prognostic value of CAFs markers and CAFs derived factors. These findings include but are not limited to FAP expression in CAFs being related to reduced survival time in non-small cell lung cancer (NSCLC; Liao et al., 2013), podoplanin expression in CAFs related to reduced recurrence-free survival in NSCLC (Ito et al., 2012; Ono et al., 2013), PDGFRβ expression in CAFs related to reduced cancer-specific survival in breast cancer patients (Paulsson et al., 2009). The correlation between particular CAFs related molecular and cancer prognosis, to some extent, reflect the education process of CAFs during tumor progression. Importantly, tumor-restraining CAFs also have particular markers and derived factors, and the increased expression of such molecules in patient samples indicates a better prognosis (Table 3). The membrane protein CAV-1 is associated with cell metabolism, modulating autophagy, cholesterol distribution, fatty acid metabolism, glycolysis, glutaminolysis, and mitochondrial bioenergetics. In CAFs, CAV-1 acts as a tumor suppression protein. CAFs that lose the expression of Cav-1 undergo a series of metabolic changes and autophagy. The process of autophagy in CAFs stimulates mitochondrial activity in adjacent tumor cells by providing a critical source of energy-rich glutamine (Nwosu et al., 2016). In a retrospective study regarding breast cancer patients, the Cav-1-positive group showed 72 months of cancer-specific survival, whereas the survival time of the Cav-1-negative group was 29.5 months (Simpkins et al., 2012). Cav-1 negative tumors show increased tumor progression, metastasis, and estrogen receptor-negative genotype compared to Cav-1 positive breast cancer (Sloan et al., 2009; Witkiewicz et al., 2009; Qian et al., 2011; Simpkins et al., 2012). Asporin is another tumor inhibitor protein expressed by CAFs in breast cancer. A histopathological study on human breast cancer (*n* = 180) indicated that asporin has low expression in TNBC and HER2 + tumors, which are both aggressive breast cancer types. Survival analysis suggested that low asporin status is an independent risk factor correlated to poor outcome, whereas high asporin expression indicated a favorable outcome (Maris et al., 2015). CD271, a neurotrophin receptor, is also named as the nerve growth factor receptor (NGFR; Liang et al., 2018; Nielsen et al., 2018). The prognostic value of stromal CD271 was assessed in 31 normal pancreases and 105 pancreatic cancer (PDACs) by an IHC assay. Stromal CD271 was express mainly in CAFs, and its high expression represented a better prognosis. Results suggested CD271 + CAFs acts to restrain cancer progression. Interestingly, CD271 + CAFs predominantly existed in the areas with strong a-SMA expression in the tumor, which suggested CD271 + CAFs was a subpopulation of SMA + CAFs. Yet, how SMA + CAFs differentiated into CD271 + CAFs and CD271- CAFs is unknown, and in pancreatic cancer, the functional role of CD271 + CAFs remains to be explored (Fujiwara et al., 2012). In another PDACs related study, approximately 10% of a-SMA + CAFs showed positive Meflin mRNA expression in *In situ* hybridization (ISH) assay. The PDACs tissue samples were divided into Meflin-high (≥20% Meflin + stromal cells) and Meflinlow (<20% Meflin + stromal cells) groups. The Meflin-high group exhibited better prognosis and more differentiated histology than the Meflin-low group, which indicated that Meflin expression in CAFs correlates with a favorable outcome of human patients with PDAC (Mizutani et al., 2019).

**TABLE 3:** CAFs related protein provide favorable clinical outcomes: main findings.

References	Gene	Human cancer type	Methods and patient number	Histopathological findings
Rhim et al., 2014	Meflin	Pancreatic cancer	ISH (*n* = 71)	1 Aproximately 10% of a-SMA + CAFs show positive Meflin mRNA expression in ISH assay. 2 Meflin-high (≥20% Meflin + stromal cells) group showe prognosis and a more differentiated histology than Meflin-low (<20% Meflin + stromal cells) group
Zhang et al., 2017	Asporin	Breast cancer	IHC (*n* = 60) mRNA (*n* = 375)	1 Asporin is low expressed in TNBC and HER2 + tumors, compared with HR + tumor 2 Low asporin express indicated to poor outcome, high asporin expression indicated a favorable outcome
Ono et al., 2013	CD271	Pancreatic cancer	IHC (*n* = 105)	1 CD271 + stromal expression was mostly detected on the tumor edge and was mainly in CAFs 2 CD271 + CAFs predominantly exist in the areas with strong a-SMA expression in the tumor 3 Stromal high CD271 expression represented a better prognosis.
Wang et al., 2017	Cav-1	Breast cancer	IHC (*n* = 358)	1 Cav-1 was predominantly expressed in CAFs in breast cancer 2 Cav-1-positive breast cancer patients showed increased cancer-specific survival compared Cav-1-negative group 3 Cav-1-deficient CAFs enhanced the invasiveness of breast cancer cells.

***Abbreviations:** HR, hormone receptor; IHC, Immunohistochemistry; ISH, In situ hybridization; and TNBC, triple-negative breast cancer.*

## Single-Cell RNA-Sequencing: A New Strategy to Classify CAFs Subpopulation

The coexistence of tumor restraining and tumor-promoting abilities within the CAFs population seems to be puzzling. These contradictory results can be explained by the existence of CAFs subpopulations with opposing functions. Therefore, it is essential to classify CAFs by combining a couple of different markers to identify their biological characteristics, thereby improving their therapeutic relevance. Using Single-cell RNA-sequencing, we can examine the transcriptome of single cells to distinguish cell subpopulations inferred by the same transcriptional programs. Single-cell RNA-sequencing allows us to recognize fibroblasts subsets within CAFs by restricting analytical cell numbers (Bartoschek et al., 2018). Li H et al analyzed CAFs from human CRC by single-cell RNA sequencing. Based on TGFβ signaling gene expression, CAFs in colorectal cancer were divided into two major subtypes: CAF-As and CAF-Bs. The CAF-As showed a high expression of matrix collagen type I α2 (COL1A2), decorin, metalloproteinase 2 (MMP2). At the same time, CAF-Bs exhibited high expression of myofibroblastic markers such as αSMA, transgelin, and PDGF-α (Li H. et al., 2017). Another study applied single-cell RNA sequencing to explore CAFs from the pancreatic cancer sample. Results verified the existence of previously reported myCAF and iCAF subpopulations (Ohlund et al., 2017) and mapped the gene signatures of these CAFs subsets. Notably, in this study, a new subpopulation of CAFs named “antigen-presenting CAFs” (apCAF) was identified. This subtype CAFs expressed MHC class II (MHCII)–related genes and presented antigens to CD4 + T cells, which regulating immune response (Elyada et al., 2019). The above studies provide evidence that single-cell RNA-sequencing helps to map the gene signature and function of CAFs subpopulations. However, we are still in the beginning to take advantage of this technique to explain the origin, maker, function, and intratumoral heterogeneity in CAFs.

## Discussion

This review summarizes the primary evidence of CAFs’ protective role against cancer. Although the hypotheses of a guarding stroma against cancer are not novel, we now possess a more robust picture of CAFs possible tumor restraining reactions. The surprising results that CAFs’ depletion in transgenic mice models accelerate tumor progression raise the cautionof non-specific CAFs depletion target therapy. Theoretically, patients can benefit from a successful anti-CAFs therapy as: (1) targeting pCAFs or the pCAFs-releasing factors; (2) normalize pCAFs to NFs; and (3) reprogramming of pCAFs to rCAFs phenotype (Sahai et al., 2020). A good example of normalizing CAFs is targeting the vitamin D receptor in pancreatic cancer. Vitamin D receptor ligand calcipotriol treatment reverted CAFs activation state into a quiescent state. This lead to an increased intratumoral gemcitabine concentration and prolonged survival time (Sherman et al., 2014). Given the reversible CAFs functions and subtypes, targeting pCAFs or reprogramming of pCAFs to rCAFs remains a challenge for the field. It is critically important to identify the individual fibroblast’s state in the CAFs population and pre-existing “lineage-restricted” effects that control CAFs phynotypes (Sahai et al., 2020).

Recently, the interaction between CAFs and immune cells in the TME has been increasingly recognized. Many studies in this field conclude that CAFs inhibit host anti-tumor immunity by shaping the immune cells, such as monocytes or neutrophils, into an immunosuppressive phenotype. By contrast, limited results are showing CAFs suppress tumor progression by enhancing host immunity (Ziani et al., 2018). Based on the above evidence of CAFs’ tumor inhibition effects, we believe that a particular CAFs subtype, which can strengthen host anti-tumor immunity might exist in the early cancer stage. A more delicate co-culture system needs to established and explore the cross-talk between tumor cells, CAFs subtype, and immune cells.

Fundamentally, CAFs biology will be best understood through subtyping by biology and by function. Single-cell sequencing provides a new insight to map the functional role of distinct CAFs types. To examine the functional associations among the diverse CAFs subpopulation, we now urgently require generating computational tools for cross-platform comparison. Notably, the CAFs’ tumor-promoting or tumor-restraining status can convert to each other depending on different tumor stages. CAFs population lineage tracing provides a useful approach to demonstrate the evolution between different CAFs subset. Accordingly, we need to figure out which factors from cancer cells or other stromal cells determine the signaling pathways of pCAFs and rCAFs and maintain their phenotype. Despite the vital research value of CAFs, our development of CAFs-oriented cancer management approaches is still in the elemental stage. Shortly, we will have a deeper understanding of the genetic events and signaling changes in the evolution of CAFs. The emergence of a broadly accepted CAF molecular subtyping will better recognize CAFs’ biological behaviors in particular contexts. These novel insights will help translate CAFs biology from bench to bedside and develop new strategies to improve precision cancer therapy.

## Author Contributions

WY and BY made substantial contributions to conception and determined the final version. ZW drafted the manuscript or revised it critically for important intellectual content. YaT, YiT, and JY contributed to table editing, figure drawing, and manuscript drafting. QY contributed to revising the manuscript by literature reviewing and drafting new parts of the manuscript. All authors read and approved the final manuscript.

## Conflict of Interest

The authors declare that the research was conducted in the absence of any commercial or financial relationships that could be construed as a potential conflict of interest.

## Abbreviations

CAFs: cancer-associated fibroblasts
CAC: colitis associated-colon cancer
DNMT1: DNA (cytosine-5)-methyltransferase 1
Evs: extracellular vesicles
HCC: hepatocellular carcinoma
HGF: hepatocyte growth factor
HR: hormone receptor
IHC: Immunohistochemistry
ISH: *in situ* hybridization
NFs: normal fibroblasts
OSCC: oral squamous cell carcinoma
PDAC: pancreatic ductal adenocarcinoma
TNBC: triple-negative breast cancer.
